# Supplementary material for: The Effect of Transoesophageal Echocardiography on Treatment Change in a High-Volume Stroke Unit
Source: J Clin Med. 2021 Feb 17;10(4):805. doi: 10.3390/jcm10040805 (PMC7922802; doi:10.3390/jcm10040805)
Supplement: Supplementary file 1 [file jcm-10-00805-s001.pdf]

Supplementary Table S1. Transthoracic Echocardiography and Neurovascular Ultrasound Evaluation for Extra- and intracranial Arteries Findings According to Considered Potential Cardiac Source of Embolism in Transesophageal Echocardiography.

	**TEE** (***n*** **= 432)**	**Abnormal TEE Findings** (***n*** **= 227)**	**Normal TEE** (***n*** **= 205)**	***p***
**Transthoracic Echocardiography**				
LVEF (%)	62.9 ± 8.5%	62.8 ± 8.9	63.0 ± 7.9	0.752
LVEDD (mm)	49.3 ± 5.8	49.3 ± 6.1	49.3 ± 5.5	0.942
LA volume (ml/m^2)	31.9 ± 15.4	33.3 ± 17.9	30.3 ± 11.9	0.051
E/A	1.8 ± 3.8	1.11 ± 0.33	2.3 ± 4.8	0.478
Moderate to Severe MR—*n*(%)	1 (2.4%)	0 (0.0%)	1 (4.2%)	1.000
Non severe MR—*n*(%)	9 (21.4%)	3 (16.7%)	6 (25.0%)	0.708
Mitral stenosis—*n*(%)	3 (7.1%)	1 (5.6%)	2 (8.3%)	1.000
Aortic stenosis—*n*(%)	6 (14.3%)	3 (16.7%)	3 (12.5%)	1.000
Moderate to severe AR—*n*(%)	12 (28.6%)	5 (27.8%)	7 (29.2%)	1.000
Mechanical/bioprosthetic valve—*n*(%)	13 (31.0%)	7 (38.9%)	6 (25.0%)	0.501
**Extracranial cerebrovascular ultrasound evaluation**				
Internal Carotid Artery occlusion - *n(%)*	10 (2.3%)	3 (1.3%)	7 (3.5%)	0.203
Vertebral Artery occlusion—*n(%)*	6 (1.4%)	2 (0.9%)	4 (2.0%)	0.429
Internal Carotid Artery Stenosis—*n(%)*	26 (6.1%)	11 (4.9%)	15 (7.4%)	0.315
Vertebral Artery Stenosis—*n(%)*	11 (2.6%)	6 (2.7%)	5 (2.5%)	1.000

Data are presented as mean ± or *n* (%). AR= Aortic regurgitation; LA = left atrial; LV = Left ventricle; LVEDD = LV end-diastolic dimension; LVEF = Left Ventricular Ejection Fraction; MR = Mitral regurgitation; TEE = Transesophageal Echocardiography;.
